# Potential Biomarkers of Metastasizing Paragangliomas and Pheochromocytomas

**DOI:** 10.3390/life11111179

**Published:** 2021-11-04

**Authors:** Anastasiya Snezhkina, Vladislav Pavlov, Alexey Dmitriev, Nataliya Melnikova, Anna Kudryavtseva

**Affiliations:** Engelhardt Institute of Molecular Biology, Russian Academy of Sciences, 119991 Moscow, Russia; vladislav1pavlov@gmail.com (V.P.); alex_245@mail.ru (A.D.); mnv-4529264@yandex.ru (N.M.)

**Keywords:** paragangliomas and pheochromocytomas, head and neck paragangliomas, malignancy, diagnostic and prognostic markers

## Abstract

Paragangliomas and pheochromocytomas (PPGLs) are rare neuroendocrine tumors originating from paraganglionic tissue in many sites of the body. Most PPGLs are characterized by nonaggressive behavior but all of them have the potential to metastasize. PPGLs represent a great diagnostic dilemma as it is difficult to recognize tumors that are likely to be metastasizing; criteria of malignancy can be found both in benign and metastatic forms. This review aims to analyze the current knowledge of the nature of metastasizing PPGLs paying particular attention to head and neck paragangliomas (HNPGLs). Potential predictors of the malignancy risk for PPGLs were summarized and discussed. These data may also help in the development of diagnostic and prognostic strategies, as well as in the identification of novel potential therapeutic targets for patients with PPGLs.

## 1. Introduction

Paragangliomas and pheochromocytomas (PPGLs) are rare neuroendocrine tumors formed from paraganglionic tissue. Since 2017, PPGLs have been classified as tumors with variable potential to metastasize [1]. Metastasizing PPGLs are usually difficult to diagnose and require evidence of regional or distant metastasis. Data on diagnostic and prognostic molecular markers for PPGL malignancy are limited, and many of the proposed factors remain controversial. There is a significant gap in the understanding of tumor pathogenesis, as well as the treatment and management of patients with PPGLs. This review summarized the current findings on the potential markers for distinguishing between metastasizing and benign tumors, as well as on the prediction of aggressive behavior of PPGLs, especially of those localized in the head and neck region.

## 2. Definition, Localization, and Distribution

Paraganglia represent groups of paraneurons derived from neural crest cells during embryonic development and are divided into sympathetic and parasympathetic. Sympathetic paraganglia consist of chromaffin cells and are involved in the secretion of catecholamines (norepinephrine, epinephrine, and dopamine), while parasympathetic paraganglia consist of glomus (nonchromaffin) cells and act as chemoreceptors [2]. Sympathetic paraganglia are associated with ganglia of the sympathetic trunk, celiac, renal, adrenal, and hypogastric plexuses. The tumors that arise from the largest sympathetic paraganglia forming the adrenal medulla are called pheochromocytomas (PHEOs). Tumors developing from paraganglia outside the adrenal gland are termed paragangliomas (PGLs). Parasympathetic paraganglia include supracardiac paraganglia, paraganglia of the carotid body, middle ear, and larynx, as well as paraganglia distributed along the vagus nerve and several other smaller paraganglia [3]. These paraganglia are common throughout the body, but most are found in the head and neck area [4]. The most common sites for head and neck paragangliomas (HNPGLs) include the carotid body followed by middle ear and vagal glomus [1]. In rare cases, HNPGLs can develop at other sites of the head and neck, such as nasopharynx, nasal cavities, paranasal sinuses, larynx, thyroid gland, and orbit [5]. Approximately a third of HNPGLs can secrete catecholamines [6]. Catecholamine-secreting PPGLs are termed functional while non-secreting ones are often termed non-functional and predominantly include HNPGLs. The main epidemiological data for PHEOs and PGLs are presented in Table 1.

## 3. Metastatic Disease

Malignancy of PPGLs is defined as evidence of metastases when paraganlionic tissue is found within lymph nodes or distant organs on histologic examination. The risk of metastasizing HNPGLs can reach 19% depending on the tumor origin (mentioned above). The most common site of metastasis for HNPGLs is cervical lymph nodes (approximately 69%) [10]; distant metastatic sites are very rare and predominantly include bones, lungs, and liver [8,16,17,18]. Based on the literature, the survival of patients with metastasizing HNPGLs varies depending on the size of the studied cohort, distribution of certain tumor localizations, metastatic sites, and treatment strategies. A number of studies revealed the 5-year relative survival rate of 59.5–84% and 10-year survival rate of 53% for patients with metastasizing HNPGLs without stratification by treatment type [10,19,20]. Therein, the 5-year relative survival rates by type of metastasis were 76.8–82.4% for patients with metastasis in regional lymph nodes and 11.8–41.4% for patients with distant metastasis [10,20]. The median age at diagnosis of 44 years and an approximately equal male/female ratio were reported for patients with metastasizing HNPGLs [10,20].

The vast majority of studies were devoted to searching for markers for distinguishing between benign and metastasizing PPGLs and the prediction of the metastatic potential. They were focused on complex or distinct features of tumors, including clinical characteristics, histopathology, mutations, epimutations, gene and miRNA expression, and biochemical profile. However, reliable and accurate diagnostic and prognostic markers for metastasizing PPGLs have not been found to date. Additionally, due to the rarity of the metastatic disease, most published studies analyzed a relatively small sample size of metastasizing cases, predominantly including PHEOs and fewer PGLs. On the one hand, it is associated with a higher frequency of PHEOs compared with other PGLs, and on the other hand, not all PGLs can be surgically removed and often require preoperative embolization that excludes the possibility of performing many molecular genetic tests. The currently proposed potential biomarkers for metastatic PPGLs are described below.

### 3.1. Clinical Characteristics

Overall for PPGLs, patients with metastatic disease are younger than ones with non-metastasizing tumors at the time of diagnosis. The female/male ratio is lower for metastasizing PPGL patients than for PPGL ones. There are no differences in systolic and diastolic blood pressure, heart rate, and body mass index (BMI) between groups [21]. Extra-adrenal location of paragangliomas is more frequently associated with the risk of malignancy in comparison with intra-adrenal sites [22]. Such clinical parameter as multifocality was shown not to play a significant role in the progression of metastasizing PPGLs [15]. Local recurrence and metastasis in non-chromaffin tissues are likely to co-occur [23,24].

Tumor size and weight are often noted as potential independent predictors of aggressiveness in PPGLs. However, the data on the correlation between this tumor characteristic and the risk of malignancy remain controversial. In the study of a representative set of PPGLs, the correlation of larger primary tumor size with rapid disease progression was revealed [15]. However, none of the HNPGLs presented as part of the sample set analyzed developed metastasis. Similar results were obtained in the study of Khadilkar et al. who found a significantly larger primary tumor size in patients with metastasizing PPGLs, but only two metastatic HNPGL cases were included in the analysis [25]. Additionally, in several studies of PHEOs and PGLs, metastasizing tumors exhibited larger tumor size and/or weight than benign ones [26,27,28]. In contrast, Thompson showed no statistically significant differences in increased tumor size alone and malignancy that was also confirmed in a more recent study of patients with metastasizing PPGLs [29,30].

### 3.2. Biochemical Markers

Most sympathetic paragangliomas produce catecholamines that are metabolized into metanephrines (normetanephrine and metanephrine) and 3-methoxytyramine (3MT). Metanephrines are more sensitive biomarkers for the presence of paragangliomas than catecholamines and are also very useful for the establishment of malignancy [31]. According to the European Society of Endocrinology Clinical Practice Guidelines, the measurements of plasma or urinary metanephrines and 3MT are strongly recommended for the screening of metastasizing PPGLs [32]. Notably, this recommendation is based on the fact that after the resection of metanephrine- or 3MT-producing tumors, the increase in their levels should indicate progression (metastasis or recurrence) or new tumor development, but only on the condition that all tumor mass is completely removed. At the same time, only a few studies have investigated plasma or urine metanephrines and 3MT secretion in benign and metastasizing PPGLs. The results on the use of metanephrine concentration as a predictor of malignancy are controversial. Feng et al. reported both higher plasma and urine levels of metanephrine in metastasizing PHEOs [33]. Higher urinary metanephrine concentrations were also detected in patients with metastasizing PHEOs and PGLs than in patients with benign ones [34]. In contrast, a more recent study of Indian patients with PHEOs and PGLs revealed a lower secretion of plasma metanephrine in metastasizing cases [25]. The normetanephrine plasma level compared with the metanephrine one was found to be higher in metastasizing PPGLs [35]. Along with the increase in normetanephrine, elevated 3MT plasma levels were detected in metastasizing PPGLs, but a significant difference between metastasizing and benign tumors was shown only for 3MT [36]. High levels of 3MT are associated with *SDHB*-mutated PPGLs that are frequently characterized by the hypersecretion of norepinephrine and/or dopamine [37]. This explains the significant correlation of high 3MT levels with metastatic disease; however, these can also present in metastasizing PPGLs in the absence of *SDHB* mutations [36]. Nevertheless, measurements of 3MT concentrations after primary surgery are very helpful for the detection of tumor progression in most patients with germline *SDHB* mutations.

Among catecholamines, measurements of dopamine levels have been suggested for the discrimination of patients with and without metastases. Dopamine-secreting PPGLs were shown to be characterized by a higher metastatic potential [38]. Elevated urine or plasma concentrations of dopamine were frequently observed in metastasizing PHEOs compared with benign tumors [38,39,40,41,42,43]. Moreover, the increased excretion of dopamine is associated with a worse tumor prognosis [15,44]. However, Eisenhofer et al. revealed higher urine and plasma dopamine levels both in PHEOs and PGLs and showed that the plasma level of dopamine metabolite 3MT was a more sensitive biomarker for the detection of dopamine-producing tumors and metastatic disease than plasma or urinary concentrations of other catecholamines or their metabolites [36]. Generally, most PPGLs related to cluster 1 are characterized by noradrenergic biochemical phenotype producing noradrenalin and/or dopamine that can explain higher levels of normetanephrine and 3MT compared with metanephrine in metastasizing PPGLs [45].

PPGLs can secrete not only catecholamines, but also various neuropeptides into the circulation. Neuropeptide Y (NPY) is widely expressed in the central and peripheral nervous system and is involved in the modulation of catecholamine secretion by adrenal chromaffin cells [46]. Increased plasma concentrations of neuropeptide Y (NPY) were detected in patients with PPGLs, particularly those with PHEOs [47]. A higher plasma level of NPY was observed in patients with metastasizing tumors than in those with benign tumors [48,49]. In a case of metastasizing extra-adrenal retroperitoneal paraganglioma, the NPY plasma level was decreased after primary tumor resection; however, its concentrations progressively increased during the postoperative period, coinciding with the documentation of metastases [50]. Conversely, decreased *NPY* gene expression was found in metastasizing PHEOs compared with benign ones [51,52]. Abnormal NPY plasma levels have not been reported for HNPGLs since these tumors are predominantly non-secreting. However, IHC analysis revealed high NPY expression in carotid body tumor tissues [53]. Further study of *NPY* gene expression in tissues of benign and metastasizing HNPGLs may help to develop its potential predictive values for these tumors.

Another candidate prognostic biochemical marker, neuron-specific enolase (NSE), was proposed by several studies [49,54]. Elevated serum levels of NSE can be found in metastasizing PHEOs along with plasma NPY levels. However, NSE levels remain normal in benign tumors, which are also characterized by increased NSY concentrations. This finding indicates that the NSE serum level is more indicative for the prediction of malignancy risk.

Chromogranin A (CgA) is a well-known marker measured in neuroendocrine tumors that is commonly used for their diagnosis. PHEOs and secreting PGLs also express this protein and are characterized by diffuse positive IHC staining for CgA, while HNPGLs can have the focal expression pattern or be completely negative for CgA [55]. CgA is concentrated and stored in vesicles with other secretory peptides and is released by exocytosis from neuroendocrine cells. Elevated circulating levels of CgA have been associated with many neuroendocrine tumors as well as PPGLs [56]. The clinical sensitivity and specificity of the plasma CgA assay were close to those for metanephrines in laboratory diagnosis of PPGLs [57]. Circulating CgA was correlated with tumor mass; however, there were controversial results on its association with metastasizing PHEOs [58,59,60]. Generally, elevated plasma levels of CgA were detected in both benign and metastasizing PHEOs, but the highest concentrations were found in patients with metastases at the time of initial diagnosis. Moreover, high levels of CgA can be retained in patients with metastatic disease even after surgery on the primary tumor [59]. Hypersecretion of CgA was also observed in HNPGLs, including a metastasizing case; therefore, it can be used for the screening and follow-up of functional HNPGLs producing CgA (but not catecholamines) [61,62,63].

### 3.3. Genetic Markers

PPGLs have the highest degree of heritability among all human neoplasms [1]. Approximately 40% of PPGLs are caused by germline mutations in one of the known susceptibility genes, including *NF1*, *VHL*, *RET*, *EPAS1*, *EGLN1*, *SDHA*, *SDHB*, *SDHC*, *SDHD*, *SDHAF2*, *FH*, *TMEM127*, *MAX*, *MDH2*, *GOT2*, *SLC25A11*, *DLST*, *H3F3A*, *DNMT3A*, *MET*, *MERTK*, and *KIF1B* [64]. Somatic mutations in the susceptibility genes occur in at least one-third of sporadic PPGL cases [65].

According to The Cancer Genome Atlas (TCGA) project, PPGLs were classified into three main clusters depending on the genetic alterations and pathways involved: pseudohypoxia, kinase signaling, and Wnt signaling [66]. Kinase signaling and Wnt signaling clusters were found predominantly in PHEOs while the pseudohypoxia cluster consists of both PHEOs and PGLs. HNPGLs were not classified as their embolization before surgery did not make it possible to carry out a complete molecular analysis. The pseudohypoxia cluster (cluster 1) includes tumors with mutations in genes related to the Krebs cycle (*SDHx*, *SDHAF2*, *FH*, and *MDH2*) and a subgroup of genes, including *VHL*, *EPAS1*/2, and *EGLN1*/2. The kinase signaling cluster (cluster 2) comprises PGLs with mutations in the *RET*, *NF1*, *TMEM127*, and *MAX* genes. These tumors are characterized by activation of the PI3K/AKT/mTOR and RAS/RAF/MAPK signaling pathways [67]. Cluster 3 includes tumors with activated Wnt and Hedgehog signaling pathways. PHEOs related to this subtype are characterized by the *MAML3* fusion genes, somatic *CSDE1* mutations, and overexpression of the *CHGA* gene encoding for chromogranin A [66].

#### 3.3.1. Mutations and DNA Methylation

The prevalence of cluster 1 mutations was shown to be more than twofold higher in patients with metastasizing PPGLs than in those with benign PPGLs. In contrast, the prevalence of cluster 2 mutations was threefold higher in patients with benign PPGLs than in those with metastasizing PPGLs [21]. Among the main genetic features associated with a high risk of the development of metastasizing PPGLs is a germline mutation in the *SDHB* gene. Testing for the germline *SDHB* mutation in patients with PPGLs is recommended by Clinical Practice Guidelines [31,32]. According to a systematic review and a meta-analysis study, the pooled incidence risk of metastasizing PPGLs for the *SDHB* mutation carriers was 17% while the prevalence ranged from 13% to 23% [68]. Among the patients with HNPGLs, the reported incidence of metastasis reaches 83% in the groups of *SDHB* mutation carriers (Table 2). In the majority of PPGL cases, the loss of function of the *SDHB* gene emerged from the combination of an inactivating germline mutation as the first hit and loss of the wild-type allele through a second-hit somatic alteration, which is in concordance with Knudson’s two-hit hypothesis for hereditary tumors [69]. It appears that the loss of heterozygosity (LOH) of the *SDHB* gene is the most frequent event causing second allele inactivation. In the 18 hereditary *SDHB*-mutated tumors studied, LOH was found in 94% of the cases; one patient had a somatic unknown intronic variant in the *SDHB* gene predicted as pathogenic in silico but with no experimental evidence of its effect on the gene’s functionality [70]. These results are also confirmed by several recent studies, which have revealed LOH as the second allele-inactivating event in *SDHB*-mutated hereditary PPGLs; at the same time, no somatic point mutations have been identified on the second allele [71,72]. Alternative mechanisms of second allele inactivation such as gene promoter hypermethylation, mutation, and epimutation of other genes or regions regulating *SDHB* expression have been poorly investigated. In particular, in sporadic PHEOs without germline and somatic mutations in the *SDHB* gene, a combination of LOH and partial methylation of the gene promoter was shown, which can probably lead to gene inactivation, but it requires more detailed study [73]. Additionally, *SDHB*-mutated tumors were characterized by a hypermethylation phenotype, resulting in significant downregulation of hypermethylated genes [74,75]. On the one hand, a tumor hypermethylation phenotype can be caused by the *SDHB* gene inactivation; on the other hand, this phenotype can also lead to the loss of the second allele through the dysfunction of the *SDHB* gene expression regulators. The highest hypermethylation level found in *SDHB*-mutated tumors in comparison to PPGLs with mutations in other susceptibility genes (*SDHx*, *FH*, *VHL*, *RET*, *NF1, MAX,* and *EPAS1*), can play a functional role in a more aggressive course of the disease. Indeed, a series of studies showed distinct DNA methylation patterns related to metastasizing PPGLs. Thus, the CpG island methylator phenotype (CIMP) was found in metastasizing PPGLs and distant metastasis, indicating that global hypermethylation initially arises in the primary tumor and can predict increased metastasis risk [76]. However, high CIMP is not always associated with metastasizing PPGLs and is not necessarily sufficient for the development of metastases [75].

To date, several CpG sites significantly associated with metastasizing PPGLs that are proposed as candidate prognostic markers have been found, including a hypermethylated site in the *RDBP* gene (cg06351503, Illumina 27K) [75], a hypomethylated site in *NTRK1* (cg00626119, Illumina 27K) [88], promoter hypermethylation in the *CDKN2A* gene locus p16INK4A [89], as well as hyper- and hypomethylated sites in the *ACSBG1* (cg02119938, Illumina 450K) and *MAST1* (cg26870725, Illumina 450K) genes, respectively [90] (a complete list of the identified CpG sites is provided in the studies cited). However, CpG hypermethylation in *RDBP* could not be fully reproduced in more recent studies [88,90]. It should be noted that all the published studies analyzed cohorts with a predominance of PHEOs and PGLs; metastasizing HNPGLs have not been studied separately.

The germline mutation in the *SDHD* gene was also reported in metastasizing PPGLs; however, the risk of metastasis development in *SDHD* mutation carriers is significantly lower than in those with an *SDHB* mutation [78]. *SDHD* mutations are more frequently associated with HNPGLs and multiple tumors [86,91,92]. The pooled risk of incidence and prevalence of metastasizing PPGLs for *SDHD* mutation carriers was estimated as 8% and 3%, respectively [68]. The incidence risk of malignancy for patients with *SDHD*-mutated HNPGLs reaches 22.7% (Table 2). The highest incidence risk of malignancy (100%, 4/4) was observed among patients with HNPGLs from the Dutch population; at the same time, no variants were found in the *SDHB* gene [84]. All these patients carried a founder mutation in the *SDHD* gene. Thus, this higher association of the *SDHD* mutation with malignancy compared with *SDHB*, which was found in most studies, can be explained by characteristics of the Dutch population.

Abnormalities in *TP53*, a well-known tumor suppressor gene, have been found both in benign and metastasizing PPGLs [93,94,95,96,97]. The study of Yoshimoto et al. revealed a high frequency of *TP53* gene variants in Asian patients with multiple and metastasizing PHEOs [94]. Part of these tumors showed nuclear p53 overexpression indicating functional mutations in the *TP53* gene and its potential participation in tumor progression. However, Petri et al. reported opposite results; it was shown that despite the loss of the p53 locus on 17p in many benign and metastasizing PHEOs, no mutations or rare positive nuclear protein immunostaining were identified in association with metastatic disease [96]. Several cases reported *TP53* mutations occurring together with pathogenic variants in other susceptibility genes in a multiple HNPGL (*TP53* and *SDHD*) [98] and a metastasizing PHEO (*TP53* and *SDHB*) [99]. In addition, a recent study revealed an antitumor effect of wild-type *TP53* upregulation in PC12 cells through the induction of cell cycle arrest and apoptosis [100]. Based on these results, it seems that *TP53* gene abnormalities can occur in the worst disease presentation but possibly play a supporting role in tumor progression.

Several studies reported germline mutations in other susceptibility genes for PPGLs, such as *FH* [101], *SLC25A11* [102], and *MDH2* [103], which were associated with aggressive tumor behavior. These genes are classified as cluster 1 TCA cycle-related associated with the pseudohypoxia subtype of PPGLs [104]. Moreover, tumors with mutations in these genes were clustered together with *SDHx*-mutated tumors demonstrating similar hypermethylation profiles as mentioned above [74,102,103,105]. This phenotype seems to be involved in tumor progression and mutations in the *FH*, *SLC25A11*, and *MDH2* genes along with *SDHB* and *SDHD* mutations can be considered to be a risk factor for PPGL malignancy. However, mutation frequency in these genes is rare and accounts for less than 1% [101,102,106]. Notably, alterations in *FH* and *SLC25A11* were found in HNPGLs but in non-metastasizing tumors [101,102,106].

Germline mutations *SDHC*, *NF1*, *VHL*, and *RET* were also reported in association with metastasizing PPGLs. Patients with *NF1* and *VHL* mutations predominantly develop PHEOs [107], including metastasizing tumors [108,109,110,111]. *RET* and *SDHC* mutations, in turn, can also occur in patients with HNPGLs [91]. *SDHC* variants were revealed in metastasizing PGLs [112,113,114], including HNPGL [85], while *RET* mutations were not associated with the aggressive behavior of HNPGLs but were detected in metastasizing PHEOs and other extra-adrenal PGLs [115,116]. Nevertheless, only a few cases reported metastatic disease in patients with mutations in these genes, and data are limited to adequately assess the risk of malignancy. Notably, germline deletion of another susceptibility gene, *MAX*, associated with the loss of gene expression was detected in a case of a metastasizing PHEO [117]. This could lead to deregulation of MYC transcriptional activity promoting tumorigenesis and metastasis [118].

*ATRX* is a frequent somatically mutated gene in PPGLs. Somatic *ATRX* mutations were revealed in tumors without any variants in the known susceptibility genes or those with inherited mutations in *SDHB*, *SDHD*, *VHL*, and *NF1* [119]. The very rare co-occurrence of somatic *ATRX* and *IDH1* mutations was found in one patient with retroperitoneal paraganglioma [120]. The most frequent *ATRX* alterations have been observed in *SDHx*-mutated tumors, including metastasizing PPGLs [119]. Moreover, several studies showed that the somatic *ATRX* variant occurring with the *SDHB* mutation and/or *TERT* overexpression was an indicative marker of metastasizing tumors [121,122]. An important role of *ATRX* and telomere maintenance mechanisms during tumor progression was also confirmed by the presence of alternative lengthening of telomeres (ALT) in *ATRX*-mutated metastasizing PPGLs [123].

Except for *ATRX*, somatic mutations in the *MYCN*, *MYO5B,* and *VCL* genes were found in patients with metastasizing PPGLs [124]. The comprehensive molecular profiling of PPGLs confirmed the relationship of somatic mutations in *ATRX* with metastatic disease and revealed additional clinical outcome markers such as somatic mutations in *SETD2* and the *MAML3* fusion gene. The latter had a strong association with the activation of Wnt signaling in PPGLs regulating many cellular processes, the alterations of which can promote more aggressive tumor behavior and metastasis [66].

Based on the same TCGA exome data for metastatic and non-metastasizing PPGLs, Such et al. revealed somatic mutations in additional genes (*RP11-798G7.5*, *HERC2*, *TGDS*, *TRHDE*, *FKBP9*, and *BMS1*) significantly associated with metastatic tumors [125]. Finally, the most recent study of 15 aggressive PPGLs identified somatic variants with higher frequency in the *SETD2*, *NF1*, and *HRAS* genes [126].

#### 3.3.2. Transcriptome Alterations

The search for candidate genes with altered mRNA and/or protein expression predicting the metastatic potential for PGLs has been performed in many studies. However, most of them have investigated PPGLs, excluding HNPGLs, as they often undergo preoperative embolization. Large-scale analysis of gene expression profiles of benign and metastasizing PHEOs has been conducted by several research groups beginning in 2006. Brouwers et al. reported 636 differently expressed genes (DEGs) between benign and metastasizing PHEOs without any considerations [127]. The majority of the following studies found a significantly lower number of DEGs that could discriminate benign and metastasizing PHEOs and, notably, most genes were predominantly downregulated in metastatic tumors compared to the benign ones [52,128,129]. Among the genes with decreased expression are those related to the neuroendocrine cell phenotype and function, indicating that cells may become less differentiated during tumor progression [52,125,128]. On the other hand, a set of overexpressed genes could include those involved in the cell cycle, cytoskeleton regulation, Wnt signaling, c-Myc and RAS activation, and several cancer-related pathways, the deregulation of which is associated with cell survival, proliferation, growth, and motility [125,129,130]. The transcriptome analysis of the TCGA and “COrtico et MEdullo-surrénale: les Tumeurs Endocrines” (COMETE) public data revealed similar significantly altered processes (cell proliferation, signaling, and metabolism) between metastasizing and benign PHEOs [131,132]. Such et al. performed gene expression profiling with the view of identifying diagnostic markers for metastasizing PPGLs [130]. They found five differently expressed genes (*CFC1*, *FAM62B*, *HOMER1*, *LRRN3*, *TBX3*, and *ADAMTS*) that are highly associated with metastasizing PHEOs in combination (are under the ROC curve (AUC) of 0.96). A higher metastatic potential was detected in PPGLs with overexpression of the *HIF1A* and *VEGF* genes [133]. Additionally, a new candidate marker of the malignancy risk, the *PNMT* gene, was identified among the DEGs found between metastasizing and benign PPGLs of Korean patients [132].

MiRNAs represent an important class of molecules and could be used as diagnostic and prognostic biomarkers in several cancers [134]. However, miRNA expression in PPGLs has been poorly investigated, especially in association with metastatic potential. The first study of the miRNA expression profiling in benign and metastasizing PHEOs revealed 18 differently expressed miRNAs and showed separate clusterization of these tumors [135]. From those, only two miRNAs, miR-483-5p and miR-15a, were validated as potential diagnostic and prognostic markers. A low level of miR-15a and high expression of the *IGF2* gene in combination was shown to have high diagnostic accuracy for the prediction of metastatic tumors (area under the ROC curve of 0.9) when the overexpression of miR-483-5p was associated with worse disease-free survival. The upregulation of miR-483-5p in metastasizing PHEOs was confirmed by another study, in which two additional miRNAs, miR-101 and miR-183, were identified as related to *SDHB* mutations and aggressive tumor behavior [136]. Additionally, the level of miR-101 was found to be higher in metastasizing PHEOs with *SDHD* mutations indicating that *SDHB*- and *SDHD*-mutated metastasizing tumors can harbor similar molecular phenotypes [137]. The overexpression of miR-210 was shown in the tumor tissue of metastasizing PPGLs and also observed to be associated with *SDHx/VHL*-related tumors with pseudohypoxic signature, which was expected as this miRNA is a target of HIFs and is consistently induced under hypoxia [66,138,139]. Opposite results were reported by Ruff et al. who showed low serum levels of miR-210 in patients with metastasizing PPGLs [140]. A more recent study identified a set of six overexpressed miRNAs (miR-21-3p, miR-183-5p, miR-182-5p, miR-96-5p, miR-551b-3p, and miR-202-5p) associated with metastatic potential in PPGLs and proposed a good predictive model of metastasis based on the tissue or serum level of miR-21-3p and miR-183-5p (area under the ROC curve of 0.80), the predictive value whereof was increased by adding the *SDHB* status (AUC of 0.84) [141].

Telomerase is another important tumor marker. This enzyme maintains telomere ends and regenerates the telomere structure. Normal somatic cells have a limited amount of cell division (Hayflick limit) that depends on the shortening of telomeres [142]. In the vast majority of somatic cells, telomerase is silent and allows cells to reach replicative senescence. The reactivation of telomerase activity can promote continuous cell growth and is closely related to malignancy. Most cancers have high telomerase activity or an active ALT pathway [143]. Telomerase activity and high expression of different components of telomerase complexes were found to be associated with metastasizing PHEOs [144,145]. However, the expression of telomerase complex components can have different abilities to predict metastatic potential. Thus, TERT RNA expression has been observed in all the metastasizing PHEOs studied, but it can also be detected in benign tumors [145,146]; hTERT expression was characterized by 100% sensitivity and better specificity than that of TERT mRNA, and was correlated with high telomerase activity [145]. Telomerase activity alone has been detected both in metastasizing and benign PHEOs [144,147,148,149]. A study by Luo et al. revealed not so good correlation results and showed elevated TERT mRNA expression detected in five out of the seven tested metastasizing PHEOs and a small number of benign cases correlating with hTERT expression [150]. Mechanisms resulting in aberrant hTERT expression in PHEOs remain unclear; however, several studies have reported promotor gene mutation [151], hypermethylation [152], and structural rearrangements [153]. Interestingly, a *TERT* mutation was more frequently found in *SDHx*-mutated tumors, but not in PPGLs [154]. This could be explained by very rare *TERT* mutations in PPGLs; however, we observed *TERT* overexpression in *SDHB*-mutated tumors with *ATRX* variants (described above) that may indicate an additional mechanism of telomerase activation.

### 3.4. Histopathological Markers

The majority of histopathological criteria were considered to be weakly correlated with metastasis in PPGLs, unlike in other malignant neoplasms [155,156,157]; research results are still controversial and depend on the analyzed cohort. In addition, based on the combination of different histological criteria, several grading systems for assessing the malignant potential of PPGLs have been proposed.

#### 3.4.1. Grading Systems

Thompson proposed an adrenal gland scaled score (PASS) method that may assist in identifying potentially aggressive PHEOs based on a complex of histological features [29]. The weighted PASS includes twelve different histomorphological parameters, and each of them has a number of points assigned. According to Thompson’s study, a PASS ≥ 4 identified tumors with malignant histology, but not all the tumors developed metastatic disease (at least 10 years of follow-up information). A PASS < 4 accurately indicated benign histology and benign clinical outcomes. Similar results were obtained in several studies, in which all the metastasizing PGLs and part of benign tumors showed PASS ≥ 4, and all the benign neoplasms had PASS < 4 [30,158,159]. Although the PASS system identifies tumors with aggressive biological behavior, it has some weaknesses and cannot be used as a reliable tool for predicting malignancy: (1) PASS components can have different interpretations among expert pathologists; (2) too many tumors are defined to have malignant potential; (3) the method was developed only for PHEOs, but not for paragangliomas of other localizations [160]. However, the PASS system may help with the initial reservation of potentially metastatic tumors. Moreover, the use of PASS score ≥ 6, as well as its combination with other prognostic factors, such as the high cellular proliferation marker Ki-67 [161], size and weight of the tumors [26], low number of S-100-positive cells [162], low intra-tumoral aromatic L-amino acid decarboxylase (AADC) expression [163], and low expression of chromogranin B (CHGB) [164], can improve the definition of tumors with malignant potential.

In 2014, Kimura et al. established a grading system for adrenal pheochromocytoma and paraganglioma (GAPP) to overcome the limitations of the PASS system [165]. The GAPP system was developed both for PHEOs and PGLs and is based on not only histological parameters (histological pattern, cellularity, comedo necrosis, and vascular/capsular invasion), but also on immunohistochemical (IHC) markers (Ki67 index) and biochemical profiles (catecholamine-secreting type). The GAPP classifies tumors into three groups using a scoring system: well-differentiated (WD), moderately differentiated (MD), and poorly differentiated (PD) tumors, among which MD and PD tumors are characterized by high metastasis risk and show progressively worse survival. More recently, it was shown that a combination of some GAPP parameters with a loss of SDHB IHC staining (M-GAPP), which is frequently observed in metastatic tumors, can better predict the metastatic potential of PPGLs [166]. A comparison of the PASS and the GAPP revealed that both systems have excellent sensitivity to malignant disease, but low specificity. This indicates that the models could be used to rule out tumors with malignancy risk rather than for the identification of those without it [167].

#### 3.4.2. Immunoreactivity

Immunohistochemistry is a standard method for the diagnosis of tumors that allows certain markers associated with specific tumor characteristics to be visualized. IHC plays an important role in the differential diagnosis of PPGLs, detection of hereditary disease, and estimation of tumor metastatic potential [168]. Several studies have reported potential IHC markers for distinguishing between metastasizing and benign PPGLs.

Currently, the *SDHB* mutation status is the best predictor for metastasizing PPGLs that can be immunohistochemically detected as negative or weak diffuse SDHB staining [169]. Nevertheless, changes in SDHB expression have been shown in tumors with mutations in any *SDHx* genes; therefore, this method can be used as the initial step for the identification of *SDHx*-mutated tumors with following genetic testing [170,171]. Patients with *SDHB* and *SDHD* (to a lesser extent) mutations can be regarded as having a risk for malignancy and warrant closer follow-up. Importantly, this IHC marker is useful for PPGLs of all localizations, unlike other ones that are predominantly validated for PHEOs. A similar approach to the prediction of malignant disease based on the identification of the *SDHx* gene mutation status is the evaluation of the succinate-to-fumarate ratio using liquid chromatography–mass spectrometry [172].

The Ki-67 protein is another important biomarker of tumor progression used in grading systems and prognosis prediction for several types of cancer [173]. It is also included in the pathological grading system GAPP for the estimation of metastatic potential in PPGLs. PPGLs usually have low proliferation activity with the Ki-67 score varying from 0% to 2%. However, elevated proliferation activity (over 2%) was observed in metastasizing PHEOs and PGLs [27,28,174]. Moreover, a series of studies have reported metastasizing PPGLs with the Ki-67 index of more than 4% [26,175]. Nevertheless, metastatic tumors can also have proliferation activity up to 2%, indicating the high specificity but low sensitivity of the method [158,176]. Recent research by Guo et al. showed an association between the Ki-67 index and the programmed death ligand 1 (PD-L1) expression in PPGLs [177]. Although PD-L1 expression was not significantly correlated with the presence of distant metastases, PD-L1 positivity in tumor cells with high Ki-67 may indicate that cells acquire the ability to escape the immune system, contributing to tumor growth, invasion, and metastasis [177,178]. The association of tumor progression with immune evasion in PPGLs was confirmed by the fact that almost half of the metastasizing PPGLs expressed PD-L1 or PD-L2 [178]. Additionally, the TCGA project study found a positive correlation of the Ki-67 index with metastasizing PPGLs and its highest expression in *MAML3* fusion-positive tumors related to the Wnt signaling cluster. This indicates that the activation of the Wnt signaling pathway can promote tumor cell proliferation and progression of paragangliomas [66].

Sustentacular (type II) cells are usually present in paragangliomas and surround chief (type I) cells forming a pattern of small nests (“Zellballen”). These cells are positively stained with the S-100 protein and the glial fibrillary acid protein (GFAP). In metastasizing HNPGLs, absence of S-100 and GFAP staining has been predominantly observed [179,180]. No sustentacular cells were observed in the primary tumor and metastasis using light and electron microscopy in a case of metastasizing carotid paraganglioma [181]. Depletion in the density of sustentacular cells was also reported for metastasizing PHEOs and PGLs [26,28,162].

Studies on the expression of heparanase-1 (HPSE1) as a candidate marker for metastasizing PPGLs have attracted great interest. This enzyme participates in extracellular matrix degradation and remodeling and is involved in cell signaling and regulation of gene transcription. HPSE1 expression normally presents in limited tissue types and is strongly regulated to prevent nonspecific tissue damage. However, almost all tumors acquire the ability to overexpress HPSE1 that promotes tumor growth and metastasis [182]. Using the IHC method, the expression of HPSE1 was validated in metastasizing and benign PHEOs in two studies [183,184]. In both studies, the positive staining of HPSE1 was found in approximately 80% of the metastasizing cases, but it was also observed in approximately one-third of the benign tumors. Similarly, cyclooxygenase-2 staining was observed in 23.68% of the benign versus 83.33% of the metastasizing cases. Employment of both HPA-1 and Cox-2 staining combined has a positive predictive value of malignancy of 0.72 [184]. Results on the stronger expression of COX2 in metastasizing PHEOs were also demonstrated in several other studies [33,185,186]. The correlation between HPSE1 and COX2 expression may indicate a possible combined role of HPSE1 and COX2 in tumor progression, particularly via the promotion of angiogenesis [184].

Another potential marker for malignancy in PPGLs, galectin-3 (LGALS3), was proposed in several studies [186,187,188]. LGALS3 is involved in numerous biological processes, such as cell proliferation, apoptosis, cell adhesion, and immunity, and plays an important role in tumor progression [189]. Previously, LGALS3 was reported as the IHC marker of thyroid malignancy [190], and recently, its overexpression was shown to be associated with the aggressive behavior of PPGLs (predominantly PHEOs).

Many years ago, an interesting prognostic model for metastasizing PHEOs was proposed, which is based on scores calculated from the IHC staining of five neuropeptides ([Leu^3^]-enkephalin, [Met^3^]-enkephalin, somatostatin, pancreatic polypeptide, and vasoactive intestinal polypeptide) and the patient’s sex [191]. The decrease in the number of positive cells for these neuropeptides was frequently observed in metastasizing PHEOs. This model correctly classified 90% of the metastasizing cases and 93% of the benign tumors and could be a promising marker for malignancy. However, there have been no further studies validating these results. Several other possible IHC markers have been reported in several studies; these are vascular endothelial growth factor (VEGF) [33], mevalonate diphosphate decarboxylase (MVD) [33], N-cadherin (NCAD) [192], calsequestrin 2 (CASQ2) [129], insulin-like growth factor 2 (IGF2) [135], phenylethanolamine N-methyltransferase (PNMT) [132], NME/NM23 nucleoside diphosphate kinase 1 (nm-23) [186], snail family transcriptional repressor 1 (Snail) [188], insulin-like growth factor 1 receptor (IGF1R) [188], heat shock protein 90 (HSP90) [193], signal transducer and activator of transcription 3 (STAT3) [193], and pyruvate dehydrogenase kinase 1 (PDK1) [194]. However, data are limited and require validation in a large cohort of patients.

## 4. Conclusions

**Figure 1:**
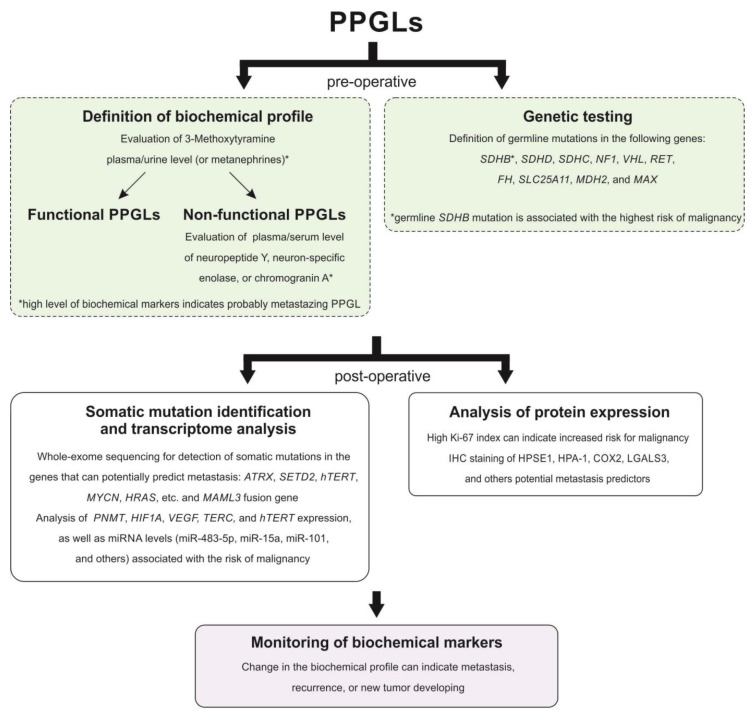
Possible scheme for the diagnosis and follow-up of metastasizing PPGLs.

Prediction of malignancy for PPGLs is a great challenge. Many scientists have been searching for specific tumor features associated with malignancy risk in PPGLs for several decades. However, most of the published works have severe limitations, including small sample size, the rarity of metastasizing cases, tumor sets with a predominance of PHEOs, and lack of (or short) follow-up. Thus, although many parameters have been suggested as potential predictive factors of malignancy, the majority of them remain controversial. Nevertheless, a combination of different markers allows an increase in diagnostic accuracy for the identification of malignant potential in PPGLs. In our opinion, the main metastasis predictors for paragangliomas of all localizations are as follows: germline mutation in the *SDHB* gene, high Ki-67 index, and high plasma level of 3-methoxytyramine. Figure 1 displays a probable algorithm for the diagnosis and follow-up of metastasizing PPGLs. The main potential markers for differentiating metastasizing and benign PPGLs are summarized in Table 3.

## Figures and Tables

**Table 1:** Epidemiological data for PPGLs.

Parameter	HNPGLs			PHEO	Other Extra-Adrenal PGLs
	CPGL	MEPGL	VPGL		
Mean age at diagnosis	40–50 * [7,8]	55 [4]	41–47 [4]	40–50 [9]	40–50 [9]
Female/male ratio	2:1–8:1 ** [10,11]	3:1–9:1 [12]	2:1–8:1 [12]	1:1 [9]	1:1 [9]
Multifocal cases, %	10–25 [1]	10–50 [13]	10 *** [14]	8 [15]	33 [15]
Metastatic cases, %	4–6 [12]	2 [12]	16–19 [10,12]	10 [9]	2.5–50 [9]

CPGL, carotid paraganglioma, MEPGL, middle ear paraganglioma, VPGL, vagal paraganglioma. * Metastatic cases are characterized by a 10-year earlier age of diagnosis. ** The ratio is higher in populations living at high altitudes under hypoxic conditions. *** Incidence of multiple paragangliomas increases to 30–40% in patients with a positive family history.

**Table 2:** Data on the incidence of patients with metastasizing HNPGLs carrying mutations in the *SDHB* and *SDHD* genes.

Mutated Gene	Incidence of Patients with Metastatic Disease (n_metastasizing_/n_mutated_) *	Reference
*SDHB*	7/13 (53.8%) 3/10 (30%) 5/6 (83%) 5/18 (28%) 3/9 (33%) 1/8 (12.5%) 3/54 (5.6%) 0/8 (0%) 1/5 (20%)	Boedeker et al. [77] Neumann et al. [78] McCrary et al. [63] Chen et al. [79] Donato et al. [80] Hong et al. [81] Rijken et al. [82,83] Hensen et al. [84] Papaspyrou et al. [85]
*SDHD*	0/6 (0%) 2/25 (8%) 0/10 (0%) 0/45 (0%) 5/22 (22.7%) 1/28 (3.6%)	McCrary et al. [63] Benn et al. [86] Neumann et al. [78] Boedeker et al. [77] Papaspyrou et al. [85] Mannelli et al. [87]

* Only studies with a sample size of mutated tumors of five or more patients were included.

**Table 3:** The main markers related to metastasizing PPGLs.

Potential Marker	Characteristics Associated with Malignancy
Histopathological markers	
Grading system for adrenal pheochromocytoma and paraganglioma (GAPP)	Well-differentiated and moderately differentiated tumors
Tumor size and weight	On average, larger than 10 cm and more than 500 g
Adrenal gland scaled score (PASS)	≥4
Ki-67 proliferation index	>2%
Sustentacular cells	Cell density depletion or absent
Galectin-3 (LGALS3)	Increased expression detected using IHC staining
Succinate dehydrogenase complex subunit B (SDHB)	Negative or weak diffuse IHC staining
Heparanase-1 (HPSE1)	Positive IHC staining
Cyclooxygenase-2 (COX2)	
Genetic markers	
Succinate dehydrogenase complex subunit B (SDHB)	Germline mutation
Succinate dehydrogenase complex subunit D (SDHD)	
Fumarate hydratase (FH)	
Solute carrier family 25 member 11 (SLC25A11)	
Malate dehydrogenase 2 (MDH2)	
ATRX chromatin remodeler (ATRX)	Somatic mutation
Histone-lysine N-methyltransferase SETD2 (SETD2)	
Telomerase reverse transcriptase (hTERT)	
Mastermind-like transcriptional coactivator 3 (MAML3)	Fusion gene
CpG island methylator phenotype (CIMP)	High CIMP
MicroRNA miR-15a	Downregulation
Phenylethanolamine N-methyltransferase (PNMT)	
MicroRNA miR-483-5p	Overexpression
MicroRNA miR-101	
MicroRNA miR-210	
MicroRNA miR-21-3p	
MicroRNA miR-183-5p	
Telomerase reverse transcriptase (hTERT)	
Biochemical markers	
Normetanephrine and 3-methoxytyramine	Increased plasma or urine level
Neuron-specific enolase (NSE)	Increased serum level
